# Circulating Tumor Necrosis Factor Receptors: A Potential Biomarker for the Progression of Diabetic Kidney Disease

**DOI:** 10.3390/ijms21061957

**Published:** 2020-03-13

**Authors:** Maki Murakoshi, Tomohito Gohda, Yusuke Suzuki

**Affiliations:** Department of Nephrology, Juntendo University Faculty of Medicine. 2-1-1, Hongo, Bunkyo-ku, Tokyo 113-8421, Japan; maki-m@juntendo.ac.jp

**Keywords:** TNF*α*, TNF receptor, biomarker, diabetic kidney disease

## Abstract

Despite considerable advancements in medicine, the optimal treatment for chronic kidney disease (CKD), especially diabetic kidney disease (DKD), remains a major challenge. More patients with DKD succumb to death due to cardiovascular events than due to progression to end-stage renal disease (ESRD). Moreover, patients with DKD and ESRD have remarkably poor prognosis. Current studies have appreciated the contribution of inflammation and inflammatory mediators, such as tumor necrosis factor (TNF)-related biomarkers, on the development/progression of DKD. The present review focuses on molecular roles, serum concentrations of TNF receptors (TNFRs), and their association with increased albuminuria, eGFR decline, and all-cause mortality in diabetes. Experimental studies have suggested that DKD progression occurs through the TNF*α*–TNFR2 inflammatory pathway. Moreover, serum TNFR levels were positively associated with albuminuria and negatively associated with estimated glomerular filtration rate (eGFR), while circulating levels of TNFRs exhibited an independent effect on all-cause mortality and eGFR decline, including ESRD, even after adjusting for existing risk factors. However, their precise function has yet to be elucidated and requires further studies.

## 1. Introduction

A total of 850 million individuals suffer from kidney disease worldwide, with one in 10 adults possibly being at risk. Diabetic kidney disease can progress to end-stage renal disease (ESRD), a condition necessitating renal replacement therapy. Diabetic kidney disease (DKD) has been one of the most significant diseases given its relationship with both progression to ESRD and morbidity and mortality from cardiovascular diseases [1]. To prevent ESRD, identifying patients at high risk for progression of DKD at an early stage and providing intensive treatment are imperative. Highly sensitive biomarkers are required to identify such patients. In classical diabetic nephropathy, the earliest clinical sign is moderately increased albuminuria (microalbuminuria: 30–300 mg/g creatinine). However, albuminuria may be a less accurate predictor of nephropathy risk than initially thought [2]. In addition, some patients with diabetes have reduced kidney function while maintaining normo- or microalbuminuria [3].

Recently, growing evidence has highlighted the importance of inflammation in the development and progression of DKD [4]. Accordingly, increased expression of cell adhesion molecules, chemokines, and inflammatory cytokines have been observed in the kidneys of patients with diabetes. Moreover, studies have determined that tumor necrosis factor alpha (TNF*α*) is a well-known inflammatory cytokine associated with the progression of kidney disease [5]. Two types of TNF receptors (TNFRs) exist, TNFR1 and TNFR2, both of which have cleaved and solubilized extracellular domains (soluble TNFR1 (sTNFR1) and sTNFR2) [6]. sTNFR1 and sTNFR2 are not deviating enzymes appearing after cytolysis but rather appear in the blood at the initial stage of inflammation.

The present review therefore describes the role of chronic inflammation in the pathogenesis of DKD, focuses on the relationship between TNF–TNFR signaling and DKD progression, and highlights the utility of TNFRs as a biomarker.

## 2. Inflammation and Diabetic Kidney Disease

Studies have shown that hyperglycemia, oxidative stress, glycation end products, glomerular hypertension, and chronic inflammation are associated with DKD progression [7,8]. Given that the inflammation observed with DKD has been considered quite mild compared to that with classic inflammatory diseases, it has been referred to as “microinflammation” in order to distinguish it from the other classic inflammation [9].

Inflammation involves a myriad of factors, including inflammatory cytokines, chemokines, adhesion molecules, innate immunity, and transcription factors. Inflammatory cytokines, such as interleukin (IL) -1, IL-6, IL-18, and TNF*α*, have been associated with DKD through recruitment of inflammatory cells, such as monocytes and macrophages [10]. Monocyte chemoattractant protein-1 acts on monocyte–macrophage receptors to aggregate monocytes in tissues. They subsequently adhere to endothelial cells through intercellular adhesion molecule-1 and vascular cell adhesion molecule-1 and then infiltrate into the tissues [11]. Macrophage infiltration into the glomerulus and tubulointerstitium causes chronic inflammation and fibrosis. In addition to these mechanisms, hyperglycemia activates many transcription factors, including nuclear factor-κB (NF-κB), which is involved in chronic inflammation [12]. As such, aggravated microinflammation in the kidney causes expansion into the mesangial and tubulointerstitial lesion with extracellular matrix accumulation [13].

Numerous inflammatory mediators have been investigated to assess their potential utility as biomarkers and/or molecular targets for DKD [9]. Although TNF*α* has been implicated in the pathogenesis of DKD, recent clinical observational studies have suggested that TNFRs were better biomarkers of renal function decline compared to TNFα [14].

## 3. Expression and Signaling Pathway of Tumor Necrosis Factor Alpha and Tumor Necrosis Factor Receptors

TNFα is a type II transmembrane protein that exists mainly in membrane-bound form (mTNF*α*) with a relative molecular weight of 26 kDa [15]. It is cleaved by disintegrin and metalloprotease protein 17 (ADAM-17), also called TNFα converting enzyme (TACE), releasing a functional soluble form (17 kDa) into the circulation [16]. This cleaved and solubilized TNF*α* is called soluble TNFα (sTNF*α*), while that measured in circulating plasma is referred to as circulating sTNF*α* [17,18]. Both sTNF*α* and mTNFα are active as non-covalently bound homotrimers.

TNF*α* has been known to have two different receptors, TNFR1 (TNFRSF1A, CD120a, p55) and TNFR2 (TNFRSF1B, CD120b, p75), with molecular weights of 55 and 75 kDa, respectively. These two receptors are typical representatives of the TNFR superfamily (TNFRSF). The TNFRSF falls into death receptors, decoy receptors, and activating receptors. Death receptors activate the caspase cascade via the death domain (DD)-initiating extrinsic apoptotic pathway. Most activating receptors mediate intracellular signals through TNF receptor-related factors (TRAFs) [19]. TNFR1 and TNFR2 are type I transmembrane proteins characterized by cysteine-rich motifs in the extracellular domain [20]. The intracellular segments of TNFR1 and TNFR2 have no homologous sequences and activate distinct signaling pathways [15,21].

**Figure 1:**
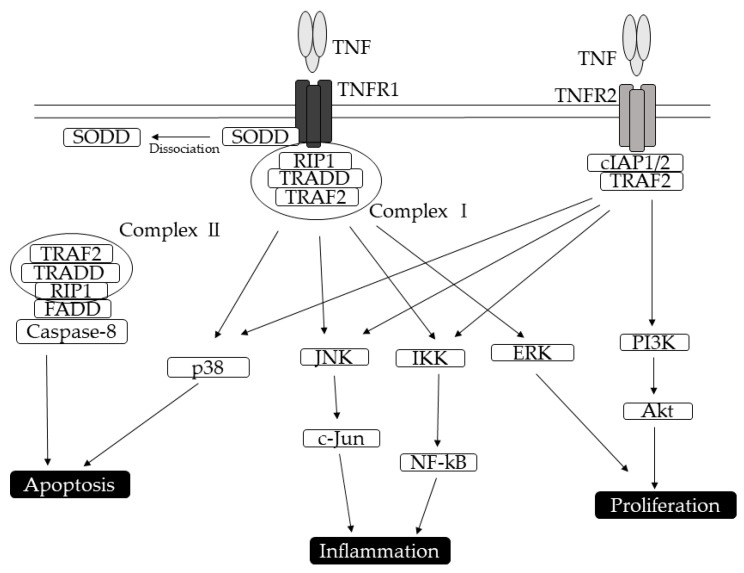
Pathways induced by tumor necrosis factor receptors (TNFRs). TNFR1 and TNFR2 activate shared and divergent signaling pathways and downstream cellular responses that lead to apoptosis, proliferation, and inflammatory mediators. The initial step in TNF-TNFR1 signaling involves the release of the inhibitory protein silencer of death domains (SODD) from TNFR1 intracellular domain. Subsequently, TNFR1 recruits distinct adaptor molecules TNFR-associated death domain (TRADD), TNFR-associated factor 2 (TRAF-2), receptor-interacting protein (RIP) at the intracellular death domain, thereby activating NF-κB-signaling, MAPK/c-Jun-signaling, and caspase-signaling. After binding of TNF*α*, TNFR2 intracellular domains recruit existing cytoplasmic TRAF2–cIAP1–cIAP2 complexes. TNFR2 activation leads to PI3K/Akt and NF-κB activation.

TNFR1 is ubiquitously expressed on almost all cell types [22]. In the kidneys, TNFR1 is mainly present in glomerular and tubular endothelial cells. The binding of TNF*α* to TNFR1 has been widely known to activate two different signaling pathways, mediate apoptosis, and regulate inflammation (Figure 1). TNFR1, which can be activated by both mTNFα and sTNF*α*, contains a homologous cytoplasmic region called DD. In the absence of a ligand, TNFR1 can interact with a cytoplasmic protein-containing DD domain called the silencer of death domain (SODD) [23,24]. The ligand-bound TNFR1 preferentially interacts with the DD-containing adaptor protein called the TNFR-associated death domain (TRADD) [25], which can replace the SODD. TRADD binds the Ser/Thr kinase receptor-interacting protein 1 and TRAF-2, forming complex I [26]. Complex I can trigger the activation of NF-κB, which functions as a transcription factor, as well as activate stress-activated MAP kinase (MAPK), p38, c-Jun N-terminal kinase, extracellular signal-regulated kinase, and other transcription factors via the MAPK3 signaling pathway [27,28,29]. Given that complex I formation is temporary, TRADD dissociates from TNFR1 and associates with the Fas-associated DD protein and caspase-8 to form complex II for caspase activation [30,31].

TNFR2, the expression of which is mainly restricted to immune and endothelial cells [32], is not usually present in the kidneys of healthy subjects [6,33]. Moreover, TNFR2 has a high affinity for membrane-bound forms of cytokines. Unlike TNFR1, TNFR2 does not have a DD in the intracellular region, suggesting the activation of a different downstream transduction pathway [34]. TNFR2 lacks the ability to recruit TRADD but can interact directly with TRAF [35]. When TNFα binds to TNFR2, the intracellular domain recruits the existing cytoplasmic TRAF-2–cIAP-1–cIAP-2 complex [15,36]. cIAPs exert ubiquitin ligase activity and can inhibit caspases and other apoptosis-inducing factors, leading to the initiation of NF-κB activation [26,36,37]. Considering that both TNFR1 and TNFR2 signals activate NF-κB, they may not be distinguished through NF-κB activation. The interaction between TNFα and TNFR2 also activates the reciprocal PI3K–Akt pathway.

As mentioned above, both TNFR1 and TNFR2 membrane receptors can also be converted to soluble forms (sTNFR1 and sTNFR2) through TACE activity [13,15]. sTNFRs inhibit TNF*α* by competing with cellular receptors for TNFα binding and perhaps also by acting as dominant-negative molecules. While low concentrations of sTNFRs enhanced TNFα action, higher concentrations abrogated the effects of TNF*α* [38]. Given that serum TNFR levels are 100–500 times higher than serum TNFα levels, circulating TNFRs might have additional functions aside from being a TNF*α*-binding protein.

## 4. Circulating Tumor Necrosis Factor Receptors: Predictive Biomarker for Diabetic Kidney Disease Progression among Patients with Diabetes

Companion studies published in 2012 by Krolewski et al. in the Journal of the American Society of Nephrology had been the first to show that circulating soluble TNFR levels predicted future renal function decline among patients with both types of diabetes over a wide variety of stages [39,40]. Patients with type 1 diabetes whose circulating TNFR2 levels were in the highest quartile had a 55% cumulative incidence of reaching stage 3 CKD, whereas those with TNFR2 levels in the lower three quartiles had a 15% cumulative incidence after 12 years of follow-up in the early stages of DKD (normal renal function and normo- or microalbuminuria). Furthermore, another study comprising patients with type 2 diabetes, including advanced DKD (stage 3 CKD and/or macroalbuminuria), showed that patients with TNFR1 levels in the highest quartile had a nearly 80% cumulative incidence of progressing to ESRD after 12 years of follow-up, whereas those with TNFR1 levels in the lowest three quartiles had <20% cumulative incidence. Thus, circulating TNFRs levels have been considered useful predictors for renal function decline after adjusting for relevant factors, such as baseline estimated glomerular filtration rate (eGFR), albuminuria, and hemoglobin A1c. Although circulating TNFRs levels also predicted cardiovascular and all-cause mortality, its predictive ability for such conditions was weaker than that for ESRD. The same study group showed that circulating TNFR2 levels also predicted the risk for ESRD among patients with type 1 diabetes and macroalbuminuria [41]. The rate of eGFR loss was especially steep among patients with increased TNFR2 and hemoglobin A1c levels.

Among patients with classical type 1 diabetes, elevated albuminuria (macroalbuminuria) has been considered to precede the development of decreased GFR (i.e., the onset of microalbuminuria leads to macroalbuminuria, followed by progressive GFR decline, and eventually ESRD). However, one study first explicated the novel model that significant GFR decline precedes the stage of microalbuminuria (*n* = 86, 35%) or had already started from the stage of normoalbuminuria (*n* = 28, 10%) in the subset of patients with type 1 diabetes (normoalbuminuria: *n* = 286, microalbuminuria: *n* = 248) within 4–10 years of follow-up [42]. Among such patients, who were called early progressive decliners, circulating TNFRs levels were associated with early progressive renal decline (eGFRcr-cys loss ≥ 3.3%/year).

Pavkov et al. presented further evidence on the utility of circulating TNFRs by investigating the association between TNFRs levels and ESRD using a very unique cohort of American Indians (Pima Indians) with type 2 diabetes [43]. Notably, the GFR used in the aforementioned study was estimated using the urinary clearance of iothalamate and not a serum creatinine-based predictive equation. The study comprised patients with relatively good renal function (median measured GFR of 120 mL/min/1.73 m^2^, with 89% of the patients having normal renal function). However, 32% of the patients developed ESRD within a relatively short period (a median follow-up of 9.5 years), indicating the considerably high risk for progression to ESRD among such cohort. Nevertheless, both TNFR levels predicted the development of ESRD in patients with type 2 diabetes, most of whom were able to preserve renal function. Furthermore, the aforementioned study showed that elevated TNFRs levels were associated with decreased percentage of normal a endothelial cell fenestration and increased mesangial fractional volume in Pima Indians with type 2 diabetes and normal renal function, suggesting that TNFRs might be associated with early glomerular lesion in diabetes [44].

Niewczas et al. [45] recently published a study regarding TNFR, wherein they measured 194 circulating inflammatory proteins using aptamer-based proteomics analysis (Slow Off-rate Modified Aptamer scan) in multiple cohorts consisting of a discovery (advanced stage of type 1 diabetes Joslin cohort), validation (advanced stage of type 2 diabetes Joslin cohort), and replication cohort (early stage of type 2 diabetes Pima Indians cohort). Accordingly, they found that 12% of the 194 measured proteins were TNFR superfamily-related proteins. Moreover, they showed that six TNFR superfamily-related proteins, including TNFR1 and TNFR2, were strong predictors of early and late renal function, leading to ESRD, in both types of diabetes.

A number of similar studies from many reputable institutions around the world have based their study on the publication by the Joslin Diabetes Center (Table 1). Overall, the measurement of circulating TNFRs has been proven to be highly effective for predicting progression to renal function decline or ESRD among patients with diabetes. The results of a recent meta-analysis also showed a substantial relation between elevated TNFRs levels and DKD progression in more than 5000 patients with diabetes [46].

## 5. Circulating Tumor Necrosis Factor Receptors: A Predictive Biomarker for All-Cause Mortality among Patients with Diabetes or Undergoing Hemodialysis

Saulnier et al. [51] examined the association between circulating TNFR1 levels and all-cause mortality in 522 patients with type 2 diabetes and DKD (eGFR < 60 mL/min/1.73 m^2^ and/or albumin/creatinine 30 mg/mmol) using a follow-up study of Survie, Diabete de type 2 et Genetique (SURDIAGENE). Accordingly, they demonstrated that patients with TNFR1 levels in the highest quartile (15.9% patient-years) had around three times higher incidence of mortality compared to those with TNFR1 levels in the lowest quartile (4.7% patient-years) after 4 years of follow-up. Compared to the Joslin Study of the Genetics of Type 2 Diabetes and Kidney Complications, the SURDIAGENE study had a much higher incidence of mortality (Table 2), which might be attributed in part to clinical characteristics (SURDIAGENE patients were older and had lower eGFR compared to Joslin patients), although the impact of circulating TNFR1 levels on ESRD was extremely similar with both studies.

We had previously reported that elevated circulating TNFRs levels were associated with the risk for all-cause mortality among 319 Japanese patients undergoing hemodialysis [53]. Sensitivity analysis revealed no significant interaction regardless of the presence or absence of diabetes. Interestingly, patients with decreased renal function had three- to five-fold higher median circulating TNFRs levels than those with preserved renal function, suggesting that circulating TNFRs accumulate with decreased renal function. On the other hand, Carlsson et al. reported that circulating TNFRs levels did not predict all-cause mortality among 207 Caucasian patients undergoing hemodialysis [54]. As such, more large-scale studies are needed to clarify such conflicting results regarding the international difference in mortality among hemodialysis patients.

## 6. Circulating Tumor Necrosis Factor Receptor Levels among Patients with Diabetes from Different Races

Circulating TNFRs levels might differ among patients with diabetes from different races. Such differences in measured values may generally be attributed to differences in measurement method. Therefore, we compared TNFRs levels among previously published studies that used the ELISA kit from the R&D system (TNFR1: DRT100, TNFR2: DRT200). Given that circulating TNFRs levels have been reported to be associated with several factors, such as age, body mass index, albuminuria, and GFR, comparing them has been difficult. However, as shown in Table 3, circulating TNFRs levels in between Caucasian and Asian patients with diabetes do not seem to be extremely different. Moreover, studies have shown that TNFR levels were quite similar among patients with diabetes and IgA nephropathy who had comparable eGFR levels [55,56]. On the other hand, Pima Indians seem to have much higher TNFR levels than other races considering their GFR and albuminuria [43]. Obesity might be partly associated with increased TNFR levels, while Pima Indians might have had naturally high TNFR levels. Further studies are required to reveal the normal range.

## 7. Contributions of TNFR1 and TNFR2 to Diabetic Kidney Disease and Other Kidney Diseases in Animal Models

Evidence for the involvement of TNF*α* and its receptors in the progression of DKD comes primarily from studies on the streptozotocin (STZ)-induced rat DKD model [58]. TNFR:Fc is a sTNF*α* antagonist consisting of two TNFR2 extracellular domains fused to the Fc portion of human IgG1, which binds to sTNF*α* and prevents TNFα from interacting with its cognate cell surface receptors. Administration of TNFR:Fc fusion protein to STZ-induced diabetic rats reduced urinary TNF*α* excretion and sodium retention and ameliorated renal hypertrophy without affecting the metabolic profile. This action might be important because sodium retention and renal hypertrophy precede the onset of albuminuria in this model. They also showed that TNF*α* stimulated sodium uptake in distal tubule cells isolated from diabetic rats. The expressions of both TNFR1 and TNFR2 were observed in distal tubule cells. Inhibiting the TNF–TNFR signaling may limit DKD progression by preventing sodium retention. Similarly, TNF*α* inhibition with infliximab, a chimeric monoclonal antibody directed against TNFα, significantly reduced both albuminuria and urinary TNF*α* excretion in STZ-induced diabetic rats [59]. We also reported that soluble TNFR2 fusion protein, Etanercept (ETN), significantly improved kidney injury in spontaneous DKD (KK-*A^y^*) mice. Renal mRNA and/or TNF*α* and TNFR1 protein levels did not differ between DKD mice with and without ETN, although those of TNFR2 improved dramatically. Moreover, ETN treatment decreased serum sTNFR2 levels but not serum sTNFR1 levels. These results suggested that the TNF–TNFR2 pathway, but not sTNFR1, may function during kidney injury in this mouse model [60]. Few studies have utilized the mouse genetic approach to understand the role of TNFα and TNFR in the DKD animal model.

However, several reports have been available concerning TNFRs in other kidney disease models (Table 4). In a rat experimental model of anti-GBM nephritis, sTNFR1 was effective in preventing acute glomerular inflammation and crescent formation both before and after establishment of nephritis [61]. Another group reported that TNFR2-deficient mice with anti-GBM nephritis did not exhibit increased albuminuria throughout the study period [62]. On the other hand, TNFR1-deficient mice exhibited albuminuria that was largely comparable to that in wild-type mice at the end of the day despite the initial delayed onset of nephritis. This study suggested that therapeutic blockade of TNFR2 may be a promising strategy for the treatment of anti-GBM nephritis. One study using a cisplatin-induced acute kidney injury model showed that TNFR2-deficient mice had milder kidney injury compared to TNFR1-deficient mice after cisplatin treatment [63] Moreover, another study has found that among unilateral ureteral obstruction (UUO) mice, TNFR1 knockout mice had less severe renal lesions, such as collagen IV deposition, α-smooth muscle actin (*α*-SMA) matrix score, and NF-κB activity, than TNFR2 knockout mice [64]. Thus, it remains unclear which among TNFR, TNFR1, or TNFR2, is strongly associated with the development and/or progression of kidney injury.

## 8. Conclusions

Considering that DKD has been a leading cause of ESRD, identifying new clinical biomarkers and therapeutic targets to effectively prevent the progression of complications is imperative. A number of studies have indicated that inflammation plays a major role in the pathogenesis of DKD, while TNF-*α* and its receptors, TNFR1 and TNFR2, contribute significantly to the progression of DKD. In addition, results from previous studies have provided sufficient evidence to suggest that TNFRs can be a prognostic biomarker for DKD. Further research is nonetheless needed to clarify the prognostic and therapeutic roles of TNFR in DKD.

## Figures and Tables

**Table 1:** Characteristics of clinical studies, diabetic kidney disease-related outcome, and circulating tumor necrosis factor receptor levels in patients with type 1 and type 2 diabetes.

Outcome	References	Type	*n*	Included Study Patients	ACR or AER	GFR (mL/min/1.73 m^2^)	Follow Up (Year)	Findings (HR (95%CI), C Index, AUC)
ESRD	Niewczas et al. [40]	2	410	eGFR >30 mL/min/1.73 m^2^ Normo, Micro, Macro	NA	eGFR >30	8–12	TNFR1: 9.4 (2.8–31.6) TNFR2: 7.6 (2.7–21.6) Per increase of interquartile range
ESRD	Forsblom et al. [47]	1	429	eGFR <60 mL/min/1.73 m^2^: 54% Macro (100%)	NA	NA	9.4	GFR+HbA1c+DM duration (Model 1): 0.72, 0.63 Model 1+TNFR1: 0.86, 0.81 C index and AUC, respectively
ESRD	Pavkov et al. [43]	2	193	eGFR >60 mL/min/1.73 m^2^: 89% Normo (32%), Micro (37%), Macro (31%)	72 (19, 493) mg/g	120 (88, 149)	9.5	TNFR1: 1.6 (1.1–2.2) TNFR2: 1.7 (1.2–2.3) Per increase of interquartile range
Time to ESRD	Skupien et al. [41]	1	349	eGFR > 30 mL/min/1.73 m^2^ Macro (100%)	771 (471, 1377) mg/g	81 (55, 104)	5–18	TNFR2: −34.6% Per increase of interquartile range
eGFR loss >3.3%/year	Krolewski et al. [42]	1	534	eGFR > 60 mL/min/1.73 m^2^ Normo (*n* = 286) Micro (*n* = 248)	Normo: 16 (12–22) mg/min Micro: 65 (44–116) mg/min	Normo: 113 (102, 123) Micro: 112 (96, 122)	4–10	TNFR1: 2.9 (1.9–4.5) Per 200 pg/mL increase in biomarker
eGFR of >40% from baseline eGFR	Saulnier et al. [48]	2	1135	eGFR > 30 mL/min/1.73 m^2^ Normo (45%), Micro (36%), Macro (19%)	3 (1–10) mg/mmol	76 ± 21	4.3	TNFR1: 1.69 (1.47–1.95) Per 1 SD increase in biomarker
eGFR of >40% from baseline eGFR	Coca et al. [49]	2	380	eGFR > 60 mL/min/1.73 m^2^ Case (50%), Control (50%)	Case: 21 (8, 66) mg/g Control: 20 (8, 102) mg/g	Case: 87 (77, 94) Control: 90 (79, 95)	5	TNFR1: 2.4 (1.5–4.0) TNFR2: 3.2 (1.7–6.1) Per doubling in biomarker
Composite renal outcome 1	Coca et al. [49]	2	1156	eGFR 30–89.9 mL/min/1.73 m^2^ Macro (100%)	NA	NA	2.2	TNFR1: 2.4 (1.7–3.3) TNFR2: 2.0 (1.4–2.8) Per doubling in biomarker
Composite renal outcome 2	Barr et al. [50]	2	194	eGFR > 15 mL/min/1.73 m^2^	NA	NA	3	TNFR1: 3.8 (1.1–12.8) Per doubling in biomarker
Stage 3 CKD	Gohda et al. [39]	1	628	eGFR > 60 mL/min/1.73 m^2^ Micro (*n* = 275) Normo + Micro (*n* = 353)	Micro: 56 (37, 101) mg/mL Normo + Micro: 41 (24, 79) mg/mL	Micro: 133 ± 30 Normo + Micro: 129 ± 30	Micro: 10–12 Normo + Micro: 5–7	TNFR1: 2.5 (1.4–4.7) TNFR2: 3.0 (1.7–5.5) Quartile 4 versus Quartiles 1–3
Mortality	Niewczas et al. [40]	2	410	eGFR > 30 mL/min/1.73 m^2^ Normo, Micro, Macro	NA	eGFR >30	8–12	TNFR1: 1.6 (1.2–2.1) TNFR2: 1.6 (1.3–2.0) Per increase of interquartile range
Mortality	Saulnier et al. [51]	2	522	eGFR < 60 mL/min/1.73 m^2^, Micro, or Macro	29 (111) mg/mmol*	49 ± 23	4	TNFR1: 3.0 (1.7–5.2) Quartile 4 versus Quartile 1
Mortality	Carlsson et al. [52]	2	607	eGFR < 60 mL/min/1.73 m^2^: 10% Micro (10%)	0.6 (0.5–0.7) g/mol**	77 (75–78)**	7.6	TNFR1: 1.8 (1.4–2.1) TNFR1: 1.5 (1.1–1.9) Per 1 SD increase in biomarker

Data are presented as mean ± standard deviation (SD), median (quartiles or interquartile range* or Bonett–Price 95% confidence intervals**); *n*, Number of patients. ACR, albumin/creatinine ratio; AER, albumin excretion rate; AUC, area under the ROC curve; CKD, chronic kidney disease; CI, confidence interval; ESRD, end-stage renal disease; GFR, glomerular filtration rate; HR, hazard ratio; Macro, macroalbuminuria; Micro, microalbuminuria; Normo, normoalbuminuria; NA, not applicable; ROC, receiver operating characteristic curve. Composite renal outcome 1: eGFR of >40% from baseline eGFR or an absolute decrease of >30 mL/min/1.73 m^2^ if the eGFR was <60 mL/min/ 1.73 m^2^ at randomization. Composite renal outcome 2: initial >30% decline in eGFR with a follow-up eGFR of <60 mL/min/1.73 m^2^, progression to renal replacement therapy, or renal death.

**Table 2:** Incidence rate of end-stage renal disease and all-cause mortality among patients with type 2 diabetes in the Joslin and SURDIAGENE study [40,51].

		Joslin	SURDIAGENE	Joslin	SURDIAGENE
		*n* = 410	*n* = 500	*n* = 410	*n* = 522
**Outcome**		**ESRD**		**Mortality**	
TNFR1	Q1	0	0	12	47
	Q2	0	4	13	77
	Q3	6	10	26	93
	Q4	84	89	49	159
		15 (59)	18 (39)	24 (84)	89 (196)

Per 1000 person years, (.) number of events. Inclusion criteria: Joslin eGFR > 30, SURDIAGENE: eGFR < 60 and/or ACR > 30 mg/mol. Mean observation time: Joslin 12 years SURDIAGENE 4 years; ESRD, end-stage renal disease; eGFR, estimated glomerular filtration rate; ACR, albumin/creatinine ratio; Q1–Q4, quartiles 1 to 4.

**Table 3:** Circulating tumor necrosis factor receptor levels among patients with diabetes and IgA nephropathy.

References	*n*	Type	GFR (mL/min/1.73 m^2^)	GFR Estimated Method	ACR (AER) or PCR	ACR Level	TNFR1 (pg/mL)	TNFR2 (pg/mL)	Race, Patients, Cohort
Pavkov et al. [43]	193	2	120 (88–149)	Urinary clearance of iothalamate	72 (19–493)	Normo (32%) Micro (37%) Macro (31%)	2833 (2081, 4092)	4835 (3875, 6997)	Pima Indian
Pavkov et al. [44]	83	2	119 (94, 155)	Urinary clearance of iothalamate	26 (12, 127)	Normo (52%) Micro (29%) Macro (19%)	1500 (1205, 1960)	3283 (2670, 4151)	Pima Indian
Gohda et al. [39]	353	1	129 ± 30	Cyctatin C based GFR	41* (24, 79)	High normo (>15) Micro	1382 (1180, 1709)	2230 (1869, 2695)	Caucasian (94%) Second Joslin Kidney Study
Gohda et al. [39]	275	1	133 ± 30	Cyctatin C based GFR	56* (37, 101)	Micro	1345 (1156, 1598)	2161 (1732, 2673)	Caucasian (94%) First Joslin Kidney Study
Skupien et al. [41]	349	1	81 (55, 104)	CKD-EPI	771 (471, 1377)	Macro	NA	4415 (3497, 5777)	Caucasian Joslin Proteinuria Cohort
Kamei et al. [57]	334	2	72 (61, 86)	IDMS-traceable MDRD	10 (6, 17)	Normo	1401 (1175, 1725)	3036 (2558, 3828)	Asian (Japanese)
Kamei et al. [57]	171	2	69 (53, 85)	IDMS-traceable MDRD	79 (40, 152)	Micro	1630 (1418, 2092)	3522 (2841, 4570)	Asian (Japanese)
Kamei et al. [57]	89	2	55 (44, 70)	IDMS-traceable MDRD	690 (449, 1487)	Macro	2229 (1751, 2811)	4370 (3610, 5645)	Asian (Japanese)
Sonoda et al. [55]	106	NA	79 (60, 100)	IDMS-traceable MDRD	0.4** (0.2, 1.0)	NA	1412 (1264, 1807)	2963 (2483, 3758)	Asian (Japanese) IgA nephropathy
Murakoshi et al. [56]	223	NA	83 ± 29	IDMS-traceable MDRD	0.4** (0.2, 1.0)	NA	1491 (1248, 1915)	3083 (2598, 3822)	Asian (Japanese) IgA nephropathy

Data are presented as mean ± standard deviation (SD), median (quartiles). *n*, Number of patients; ACR (mg/g), albumin/creatinine ratio; *, AER (mg/min), albumin excretion rate; **, PCR (g/g), protein/creatinine ratio; IDMS, isotope dilution mass spectrometry; MDRD, modification of diet in renal disease; CKD-EPI, chronic kidney disease epidemiology collaboration; GFR (mL/min/1.73 m^2^), glomerular filtration rate; NA, not applicable

**Table 4:** Inhibitory effects of tumor necrosis factor signal in animal models.

Model Used	Methods of TNFα Antagonism	Effect of TNFα Inhibition	References
Anti-GBM nephritis rat	Soluble TNFR1	sTNFR1 prevented acute glomerular inflammation and crescent formation	[61]
Anti-GBM nephritis rat	Rat TNFα monoclonal antibody	TNF antibody reduced glomerular inflammation, crescent formation, and tubulointerstitial scarring, with preservation of renal function	[65]
Anti-GBM nephritis mice	TNFR1 or TNFR2 KO mice	TNFR1-deficient mice: less proteinuria and glomerular injury only at the early stages TNFR2-deficient mice: completely protected from glomerulonephritis at all stages	[62]
TNF administration in SLE-prone mice	TNFR1 and/or TNFR2 KO mice	TNFR1/TNFR2-double deficient mice exhibited accelerated pathological and clinical nephritis	[66]
STZ-induced diabetic rat	TNFR:Fc	TNFR:Fc reduced urinary TNF excretion, sodium retention, and attenuated renal hypertrophy	[58]
STZ-induced diabetic rat	Infliximab	Infliximab ameliorated urinary albumin and TNFα excretion	[59]
Spontaneous DKD mice	Etanercept	Etanercept improved albuminuria and decreased serum sTNFR2 levels	[60]
UUO mice	TNFR1 or TNFR2 KO mice	TNFR1 or TNFR2 deficiency resulted in significantly less NF-κB activation compared with the wild type, with TNFR1 being less than TNFR2 knockout	[64]
UUO rat	Soluble TNFR1 (PEG-sTNFR1)	PEG-sTNFR1 significantly reduced tubulointerstitial fibrosis and a progressive renal function decline	[67]
Cisplatin-induced renal injury mice	TNFR1 or TNFR2 KO mice	TNFR1 or TNFR2 deficiency protects mice from cisplatin-induced AKI. TNFR2-deficient mice developed less severe renal dysfunction compared with either TNFR1-deficient or wild-type mice	[63]

TNF, tumor necrosis factor; KO, knockout; STZ, streptozotocin; UUO, unilateral ureteral obstruction; AKI, acute kidney injury

## Abbreviations

CKD	Chronic kidney disease
DKD	Diabetic kidney disease
ESRD	End-stage renal disease
TNFα	Tumor necrosis factor alpha
TNFR	TNF receptor
